# Acid stress damage of DNA is prevented by Dps binding in *Escherichia coli *O157:H7

**DOI:** 10.1186/1471-2180-8-181

**Published:** 2008-10-15

**Authors:** Kwang Cheol Jeong, Kai Foong Hung, David J Baumler, Jeffrey J Byrd, Charles W Kaspar

**Affiliations:** 1 Department of Bacteriology, University of Wisconsin, Madison, WI 53706, USA; 2 Cellular and Molecular Biology Program, University of Wisconsin, Madison, WI 53706, USA; 3 Department of Biology, St. Mary's College of Maryland, St. Mary's City, MD 20686, USA; 4 Department of Molecular Microbiology, Washington University, St. Louis, MO 63130, USA; 5 Genome Center of Wisconsin, University of Wisconsin, Madison WI, 53706, USA

## Abstract

**Background:**

Acid tolerance in *Escherichia coli *O157:H7 contributes to persistence in its bovine host and is thought to promote passage through the gastric barrier of humans. Dps (DNA-binding protein in starved cells) mutants of *E. coli *have reduced acid tolerance when compared to the parent strain although the role of Dps in acid tolerance is unclear. This study investigated the mechanism by which Dps contributes to acid tolerance in *E. coli *O157:H7.

**Results:**

The results from this study showed that acid stress lead to damage of chromosomal DNA, which was accentuated in *dps *and *recA *mutants. The use of *Bal*31, which cleaves DNA at nicks and single-stranded regions, to analyze chromosomal DNA extracted from cells challenged at pH 2.0 provided *in vivo *evidence of acid damage to DNA. The DNA damage in a *recA *mutant further corroborated the hypothesis that acid stress leads to DNA strand breaks. Under *in vitro *assay conditions, Dps was shown to bind plasmid DNA directly and protect it from acid-induced strand breaks. Furthermore, the extraction of DNA from Dps-DNA complexes required a denaturing agent at low pH (2.2 and 3.6) but not at higher pH (>pH4.6). Low pH also restored the DNA-binding activity of heat-denatured Dps. Circular dichroism spectra revealed that at pH 3.6 and pH 2.2 Dps maintains or forms α-helices that are important for Dps-DNA complex formation.

**Conclusion:**

Results from the present work showed that acid stress results in DNA damage that is more pronounced in *dps *and *recA *mutants. The contribution of RecA to acid tolerance indicated that DNA repair was important even when Dps was present. Dps protected DNA from acid damage by binding to DNA. Low pH appeared to strengthen the Dps-DNA association and the secondary structure of Dps retained or formed α-helices at low pH. Further investigation into the precise interplay between DNA protection and damage repair pathways during acid stress are underway to gain additional insight.

## Background

The extreme acidity (~pH 2.0) within the stomach presents a formidable hurdle for bacteria whose primary niche is in the lower intestinal tract of warm-blooded animals [1]. Low pH is detrimental to microbes due to the denaturation of essential macromolecules, like proteins, and the acidification of the cytoplasm that disrupts enzymatic reactions and membrane potentials [2-5]. Human enteric pathogens, like *Escherichia coli *O157:H7 and *Salmonella typhimurium*, are able to tolerate acidic conditions for a period of time through membrane exclusion of protons, pH homeostasis systems, and the protection and/or repair of essential cellular macromolecules [6-11]. In addition to an organism's innate acid tolerance, extrinsic factors like the presence of the organism in a food as well as the composition of the food can impact survival through the gastric barrier [12].

One protein that contributes to the acid tolerance of *E. coli *O157:H7 is the DNA-binding protein in starved cells (Dps), which is expressed at low levels during late exponential growth and becomes the most abundant protein in stationary-phase cells [10,13-15]. In addition to its participation in acid tolerance, Dps plays an important role in survival during other stress, including starvation, near-UV and gamma irradiations, thermal stress, metal toxicity, and oxidative stress [14,16-18]. DNA is the common target of Dps protection regardless of the stress through physical association and/or sequestration of reactants that produce free radicals. Some of the mechanisms by which Dps protects *E. coli *from starvation and oxidative stress have been defined [17,19,20], but its precise role in acid stress tolerance has not been determined. In starved cells, biocrystals formed by Dps-DNA interactions have been observed and a protective role of these structures has been proposed [19,21]. Aside from its role in stress protection, Dps has also been implicated in gene regulation based on analyses of two-dimensional gel electrophoresis patterns of proteins from a *dps *mutant of *E. coli *and the parent strain [14]. Further, highly ordered nucleoprotein complexes capable of altering gene expression patterns are observed when Dps binds DNA in some conditions [22]. All of these observations of Dps-DNA interactions were made under circum-neutral pH conditions. Since acidification of the cytoplasm during acid stress alters the internal pH of cells, it is not clear if Dps employs these same mechanisms of protecting DNA during acid stress.

To begin unraveling the mechanism by which Dps contributes to acid tolerance in *E. coli *O157:H7, both *in vivo *and *in vitro *approaches were used to demonstrate that Dps protects DNA from acid stress damage. Acid challenge of whole O157:H7 cells resulted in chromosomal DNA damage that increased with exposure time and was more prominent in a *dps *mutant. Based on the evidence of DNA damage, a *recA *mutant was generated and was found to exhibit significantly reduced acid tolerance. *In vitro *studies demonstrated that the association of Dps with DNA protected the DNA from acid damage. Circular dichroism spectroscopy demonstrated that at low pH (2.2 and 3.6) Dps formed or maintained an α-helix conformation that is associated with Dps binding to DNA. Low pH was also observed to influence the stability of Dps-DNA complexes and restored the DNA-binding activity of heat-denatured Dps by an unknown mechanism.

## Methods

### Bacterial strains, plasmids, and primers

*E. coli *strains (Table 1) were grown aerobically in Luria-Bertani (LB) medium [23] at 37°C with shaking (150 rpm). When required, antibiotics (ampicillin, 100 μg/ml or kanamycin, 100 μg/ml) were added to the medium. Cell density of broth cultures was monitored at OD_600 _using a spectrophotometer (Beckman Coulter). The plasmids and primers used in this study are listed in Tables 1 and 2, respectively.

**Table 1:** Strains and plasmids used in this study.

Strains and plasmid	Relevant characteristics^a^	Reference or source
Strains		
DH5α	*supE44 ΔlacU169 *(Φ80 *LacZ *ΔM15) *hsdR17 recA1 endA1 gyrA96 thi-1 relA1*	Lab collection
SY327 λ*pir*	*Δ *(*lac *operon) *argE*(Am) *recA56 rpoB *λ*pir*; host for π-requiring plasmids	[26]
SM0 λ*pir*	*thi thr leu tonA lacY supE recA::*RP4-2-Tc::Mu λ*pir*, *oriT *of RP4, Km^r^	[38]
BL21(DE3)	BL21λ (DE3) under *lac *control; *ompT lon dcm*	Novagen
ATCC43895	*stxI stxII *serotype O157:H7	Lab collection
FRIK47992	ATCC43895, *dps::npt1*	[10]
FRIK4704-kcj05	ATCC43895, *ΔrecA*	This study
FRIK4704-kcj06	FRIK47992 (ATCC43895, *dps*, *ΔrecA*)	This study
		
Plasmids		
pUC4K	pUC4 with *nptI*; Ap^r^, Km^r^	[39]
pCVD442	R6K γ*ori, sacB, oriT *of RP4; Ap^r^	[24]
pKCJ0325	pET21b with *dps; *Ap^r^	This study
pKCJ0328	pCVD442 with *alas::mltB; *Ap^r^	This study

^a^Ap^r^, ampicillin resistant; Km^r^, kanamycin resistant

**Table 2:** Primers used in this study.

Primers	Sequence (5' ♦ 3'^a^)	Use
kc0305	GGCATATGAGTACGCTAAATTAGTT	Dps purification
kc0306	GGCTCGAGTTCGATGTTAGACTCGATAAACC	Dps purification
kc0308	GTTAACGTGTTGCAGCACCG	*recA *construction
kc0309	CTCAACGCCGGATTTCTCTGT	*recA *construction
kc0310	ACAGAGAAATCCGGCGTTGAGAGGTAGAGATGGTTTCCACATCC	*recA *construction
kc0311	CCGCTCAATCTGAAAGGTTCCTT	*recA *construction
kc0312	TGCTCTAGACCAGATCTCAATGTAGCGGTCG	*recA *construction
kc0313	ACATGCATGCGACAGTTTATGCCGTCGTCTTAC	*recA *construction

^a ^Underlined sequence represents engineered restriction site: CATATG – *Nde*I; CTCGAG – *Xho*I; TCTAGA – *Xba*I; GCATGC – *Sph*I.

### Acid challenge

Acid challenges were conducted at pH 2.0 as described previously [10]. Briefly, an overnight culture grown in LB (10 g Tryptone, 5 g yeast extract, 10 g NaCl) under appropriate antibiotic selection was diluted 1:10,000 into 50 ml of fresh LB medium and allowed to grow until early stationary phase (OD_600 _between 1.2 and 1.4). Cells were diluted in fresh LB (1:10) and used to inoculate (1:100) 50 ml of acid challenge medium (LB adjusted to pH 2.0 using 6 N HCl, then autoclaved for 15 min) in a 250 ml flask. At specific time points, samples were removed and plated on LB agar following serial dilution in sterile phosphate buffered saline (PBS). The number of colony forming units (CFU) was determined after 24 h of incubation at 37°C. The limit of detection for this assay is 5 CFU/ml, based on colony growth on 2 duplicate plates each inoculated with 100 μl of undiluted sample. The growth of cells and acid challenges were performed at 37°C with shaking at 110 rpm.

### Mutant construction

The suicide vector, pCVD442, was used to generate a *recA *mutant and a *dps recA *double mutant in *E. coli *O157:H7 ATCC 43895 by homologous recombination [24]. In brief, using primers shown in Table 2, the 2.7-kb PCR fragment *alaS::mltB *(*ΔrecA*), was amplified using the joint PCR method previously described [25]. The fragment was cloned into *Xba*I and *Sph*I-digested pCVD442 to form pKCJ0328. The constructed vector contains the RP4 origin of transfer (*oriT*) and is conjugally mobilized from donor cells containing the *tra *gene. The recipient strains, ATCC 43895 or FRIK 47992 (*dps*), and donor strain, *E. coli *SM10 λ*pir *with the constructed vector, were grown separately to mid-log phase in LB and conjugated to generate *recA *and *dps rec*A mutants strains, respectively [24,26]. The mutants were selected and confirmed as previously described [24].

### Whole Cell DNA damage assay

Cells were grown 5 h in LB and then challenged at pH 2.0. Samples were removed after 0 min, 1, 2, 3, and 4 h of acid challenge and chromosomal DNA extracted using a genomic DNA isolation column (Qiagen) following the manufacturer's instructions. When testing for nicked-DNA, the isolated chromosomal DNA (1 μg) was subjected to *Bal*31 nuclease (0.2 units) digestion at 30°C for 30 min in a final volume of 20 μl and reactions were inactivated at 75°C for 10 min, and then chilled on ice. DNA was analyzed following electrophoresis in a 0.8% (w/v) agarose gel with ethidium bromide staining.

### Dps purification

The *dps *gene was PCR amplified with primer pair kc0305 and kc0306. These primers contain an *Nde*I or *Xho*I restriction enzyme site, respectively, for cloning into pET21b, resulting in pKCJ0325. The DNA sequence of the cloned insert was confirmed by sequencing. C-terminal His6-tagged Dps recombinant protein was purified from *E. coli *BL21(DE3). To induce expression of Dps-His6 protein, isopropyl β-D-thiogalactoside (IPTG, Sigma-Aldrich) (1 mM) was added when the OD_600 _of the culture reached 0.6, followed by a two-hour incubation. The cells were harvested by centrifugation (10 min at 4000 × *g*) and the pellet was stored at -70°C. The cell pellet was thawed for 15 min at room temperature and resuspended in 1 ml of lysis buffer (50 mM NaH_2_PO_4_, 300 mM NaCl, 10 mM imidazole, pH 8.0). Lysozyme was added to a final concentration of 1 mg/ml and incubated on ice for 30 min. Cells were lysed by sonication (300 watts; 6 × 10 sec with 10 sec pauses between pulses). Cellular debris was removed by centrifugation (10,000 × *g*, 4°C for 20 min) and the supernatant was decanted into a clean tube. Dps-His6 was isolated from the supernatant using Ni-NTA chromatography following the manufacturer's protocol (Qiagen). The final buffer was replaced with 50 mM Tris-Cl buffer (pH 7.0) containing 50 mM NaCl using PD-10 columns (Amersham Biosciences).

### *In vitro *DNA binding and damage assays

An *in vitro *DNA-binding assay was conducted as previously described [17,27]. pUC18 plasmid DNA (300 ng) was mixed with varying concentrations of Dps in 10 mM Tris (pH 6.8), 20 mM NaCl, 0.5 mM EDTA in a total volume of 20 μl and incubated 1 h at room temperature. The Dps and DNA were mixed at the following ratios (w/w): 3:1, 17:1, and 33:1. The Dps-DNA mixtures were extracted with phenol:chloroform (1:1), the DNA precipitated with ethanol, and analyzed in 1.0% agarose gels. The DNA damage assays were performed with 300 ng of pUC18 and 10 μg of Dps. Dps-DNA mixtures were incubated 1 h at room temperature to allow association and then acidified with 0.1 N HCl to pH 4.6, 3.6, 2.6, and 2.2. The acidified Dps-DNA mixtures were incubated for 2 h at 37°C and then 10 mM Tris (pH 8.5) was added. DNA was recovered by extraction with chloroform:isoamyl alcohol (24:1), followed by 10 min at 55°C in the presence of 2% SDS, precipitated with ethanol, and resolved by agarose gel electrophoresis. For experiments using heat inactivated Dps, 10 μg of Dps was heated to 75°C for 15 min.

### Circular dichroism analyses

CD spectra were recorded with an AVIV model 202SF CD spectrometer (Lakewood, NJ) at the Biophysics Instrumentation Facility (University of Wisconsin – Madison). Spectra were collected using 0.3 mg of Dps/ml in a solution of 10 mM Tris-Cl and 10 mM NaCl (pH 6.8) in a cuvette with a 1-mm-path length at 25°C. The signal was averaged for 7 s during wavelength scans. The percent of secondary structure was calculated by the method of Chen et al. [28].

### Data analyses

Except where noted, data presented are representative of at least three independent trials. Images of ethidium bromide stained agarose gels were captured using a Kodak digital camera attached to the imaging system. The relative intensity of DNA staining in each lane was quantified using Kodak 1D Image Analysis software. Briefly, the lanes on each gel were divided into an arbitrary number of discrete steps, with intensity at each step being measured. Relative peak intensity data were exported to Excel (Microsoft, Redmond, WA) for further analysis and plotting. Averages, standard deviations, and student t-tests were performed using Excel.

## Results

### Acid-induced damage of chromosomal DNA in whole cells

**Figure 1:**
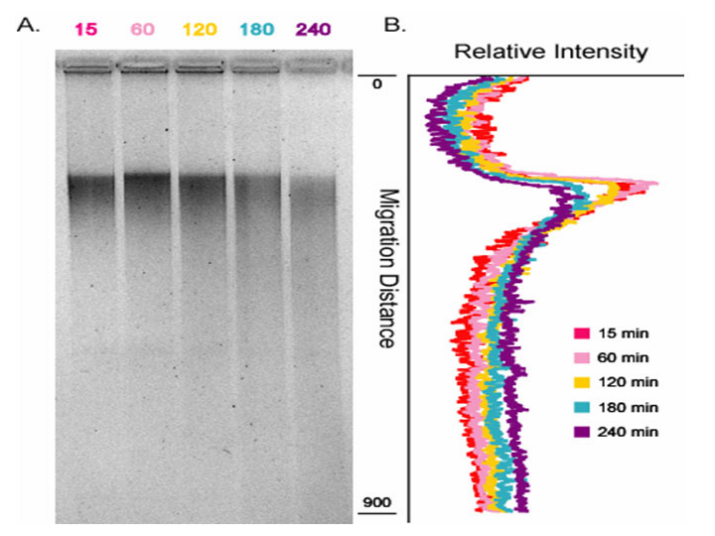
**Chromosomal DNA from acid-challenged cells**. The integrity of chromosomal DNA deteriorates with exposure of cells to acid challenge. Log-phase cells of parent strain (ATCC43895) were acid challenged at pH 2.0 for 15–240 min (time of exposure is indicated above each well). Genomic DNA was extracted, purified, and quantified before equal amounts were loaded into an agarose gel. (A) Visualization of ethidium bromide staining. (B) Relative intensity of each sample lane plotted against assigned migration distance.

To determine if acid stress results in chromosomal DNA damage, *E. coli *O157:H7 cells were exposed to acidic LB (pH 2.0) for up to 4 h, followed by extraction and examination of chromosomal DNA. At the 0 min sample point (15 min of exposure due to centrifugation step), a single, condensed band of chromosomal DNA was recovered from cells (Fig. 1). Chromosomal DNA extracted from cells that were not exposed to acidic LB also resulted in a single condensed band similar to results from the first sample point (data not shown). No significant differences were detected between cells incubated for 60 min or 120 min when compared to cells harvested at the 0-min sample point. However, with longer incubation times (180 and 240 min), the primary chromosomal DNA bands became broader, less intense, and exhibited tailing which is indicative of DNA degradation and fragmentation. The visual examination of the DNA patterns was corroborated by the densitometric measurements (Fig. 1B), where the peaks for cells incubated for 180 min and 240 min had broader bands with lower relative intensities than DNA extracted from cells incubated for shorter exposure times. Also, the decrease in the relative quantity of high-molecular weight DNA retained in the loading wells with increased time of exposure of cells to acid suggested that acid stress resulted in the deterioration of chromosomal integrity.

**Figure 2:**
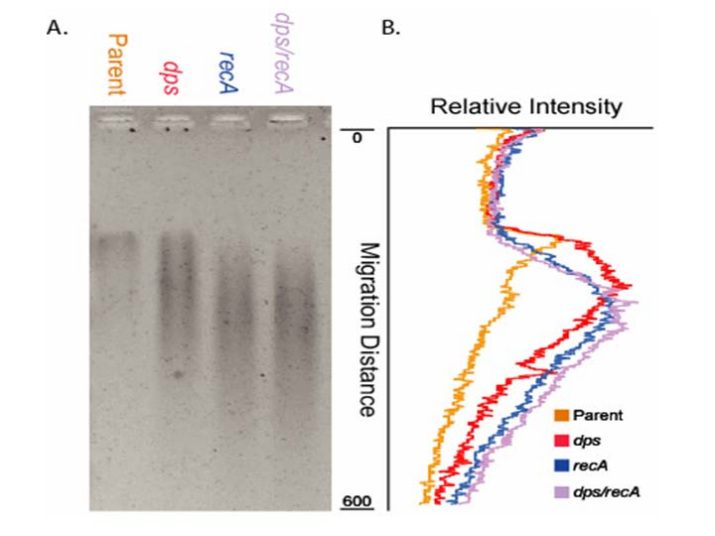
***Bal*31 digestion of chromosomal DNA from acid-challeged cells**. *Bal*31 nuclease digestion of chromosomal DNA recovered from acid-stressed cells revealed increased damage in *dps*, *recA*, and *dps recA *strains of *E. coli O157:H7*. Log-phase cells from the parent strain (ATCC43895), *dps *(FRIK47992), *recA *(FRIK4704-kcj05), and *dps recA *(FRIK4704-kcj06) were acid stressed at pH 2.0 for 2 h before genomic DNA was extracted and quantified. One micro-gram of DNA was digested with *Bal*31 nuclease and the entire digestion mixture was loaded into an agarose gel. (A) Visualization of ethidium bromide staining. (B) Relative intensity of each sample lane plotted against assigned migration distance.

The nature of the DNA damage from cells exposed to acid was examined further using *Bal*31, which cleaves DNA at nicks, gaps, single-stranded regions or other lesions of duplex DNA. The presence of strand breaks was detected in chromosomal DNA from cells exposed to acid stress. Specifically, when chromosomal DNA from parent and *dps *mutant strains were compared, the DNA from the mutant strain showed a more pronounced degradation with *Bal*31 (Fig. 2), suggesting that chromosomal DNA from *dps *mutants contained more DNA damage than the parental counterpart.

### Role of Dps and RecA in acid tolerance of *E. coli *O157:H7

Since chromosomal DNA from cells exposed to acid stress contained strand breaks, the role of RecA, which is involved in DNA repair, was examined. Using agarose gel patterns of DNA digestions with *Bal*31, the integrity of chromosomal DNA from parent, *dps*, *recA*, and *dps recA *strains that had been acid challenged were compared (Fig. 2). After exposing cells to acidic LB (pH 2.0) for two hours, the *Bal*31 cleavage of DNA from both *recA *and *dps recA *mutant strains was more extensive than either the parental or the *dps *strains, demonstrating that *recA *and *dps recA *strains had more DNA damage resulting from acid stress.

**Figure 3:**
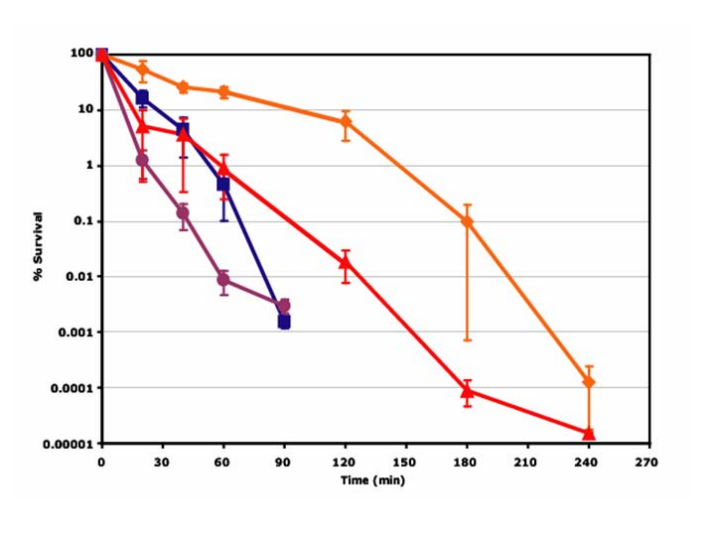
**Decreased acid tolerance in *dps*, *recA*, and *dps recA *strains**. Acid challenges of parent (◆ ATCC43895), *dps *(▲ FRIK47992), *recA *(■ FRIK4704-kcj05), and *dps recA *(● FRIK4704-kcj06) strains were performed and the percent survival for each strain was monitored over time. Averages of at least three independent trials with standard error of the mean represented by error bars are presented.

To investigate whether chromosomal DNA damage in *recA *and *dps recA *strains correlated with the survival of strains during acid stress, acid challenges were performed (Fig. 3). Since the limit of detection of the assay was 5 CFU/ml, this value was assigned to experimental conditions (*recA *and *dps recA *mutants at 90 min, and *dps *mutant at 240 min) where no survivors were detected. The rate of reduction in survival, as indicated by the slope of the best-fit line (not shown), was significantly higher in *recA *when compared to either the parental strain (*p *= 1.12 × 10^-3^) or the *dps *mutant strain (*p *= 1.34 × 10^-5^). No statistically significant difference was detected in survival during acid challenge between *recA *and *dps recA *strains. When all the time points were considered together, the rates of decline in survivability of the parent and *dps *strains did not differ significantly (*p *= 4.37 × 10^-1^). However, there were differences in the number of survivors after one hour of acid challenge (*p *= 2.15 × 10^-3^) and after 40, 60, and 120 min of acid challenge, with *p *< 0.05 at these time points. These findings link DNA damage and/or repair with a decrease in an organism's ability to survive acid stress.

### Dps binding and protection of plasmid DNA *in vitro*

**Figure 4:**
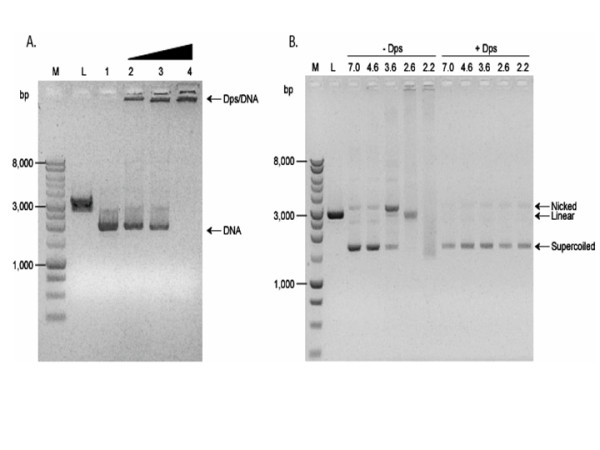
**Dps protects DNA from acid-induced damage *in vitro***. M, 1 kb DNA marker (Promega). L, *Hind*III linearized pUC18 plasmid. (A) Representative data set on determination of optimal amount of Dps required to bind supercoiled pUC18 plasmid (300 ng). Lane 1, untreated pUC18 plasmid. Lanes 2 to 4, pUC18 plasmid incubated with Dps:DNA ratio (w/w) of 3.3, 16.3, and 33.3, respectively. (B) Representative data set showing Dps protection of DNA from acid-induced damage *in vitro*. Supercoiled pUC18 DNA was either mixed with Dps (ratio 33:1, w/w, +Dps) or with control buffer (-Dps) for 1 h. The pH of the reactions was then adjusted as indicated above the wells and the samples were incubated for 2 h at room temperature. DNA was then extracted with chloroform:isoamyl alcohol (24:1) in the presence of 2% SDS, precipitated with ethanol, and resolved by agarose gel electrophoresis.

*In vitro *experiments were conducted to demonstrate conclusively that the physical association of Dps with DNA provided DNA protection from acid damage. First, the binding of Dps to DNA with varying concentrations of Dps was examined to determine the Dps:DNA ratio that bound all plasmid DNA under the assay conditions employed. The results from gel mobility shift assays showed that Dps completely bound pUC18 DNA (300 ng) at a ratio of approximately 33:1 (w/w) (Fig. 4A). Using this ratio, purified Dps and plasmid DNA were incubated in solutions of varying pH. After incubation, protein was extracted using chloroform:isoamyl alcohol (24:1) in the presence of 2% SDS and the plasmid integrity was determined by the relative abundance of super-coiled, nicked, and linearized forms of the plasmid. Results showed that plasmid incubated with Dps remained in the supercoiled form at all challenge pH values, with little detectable nicking (Fig 4B). In contrast, when Dps was omitted from the assay, the plasmid was nicked and/or linearized at pH 3.6 and at pH 2.6. At pH 2.2, the plasmid DNA was degraded when Dps was absent.

### Effect of pH on DNA binding by Dps

**Figure 5:**
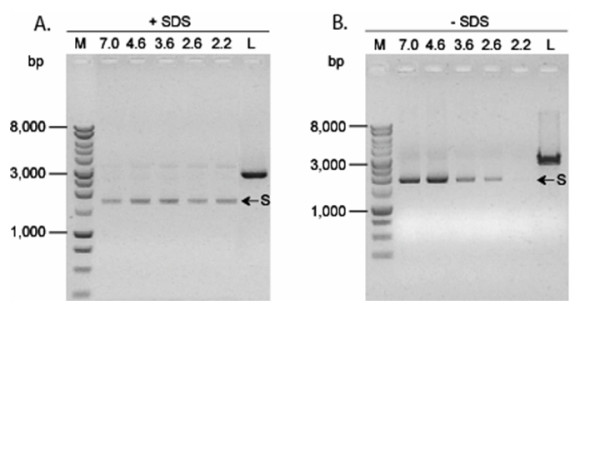
**The Dps:DNA complex is more difficult to disrupt at low pH**. Solutions containing a mixture of Dps and DNA (ratio 33:1, w/w) (300 ng total DNA) were incubated for 1 h, adjusted to the designated pH as indicated at the top of the wells, and incubated for 2 h at room temperature. DNA was extracted either with (A) with chloroform:isoamyl alcohol (24:1) in the presence of 2% SDS (+SDS) or with (B) phenol:chloroform (1:1) (-SDS), followed by precipitation with ethanol before being resolved in an agarose gel. Lane M, 1 kb DNA marker (Promega). Lane L, linear pUC18 DNA from *Hind*III digestion. The arrow points to the position of supercoiled plasmid DNA on each gel.

The amount of DNA extracted from in vitro DNA assays was influenced by pH and the extraction method employed. When denaturing extraction [chloroform:isoamyl alcohol (24:1) with 2% SDS] was used, the amount of DNA recovered was comparable regardless of the challenge pH (Fig. 5A). In contrast, extraction using non-denaturing conditions [phenol:chloroform (1:1)] yielded less DNA when Dps and plasmid DNA were incubated at pH ≤ 3.6 (Fig. 5B), and the amount of extractable DNA decreased with lower pH. At pH 2.2, DNA was barely detectable. These data suggested that the binding of Dps to DNA was influenced and enhanced at lower pH or Dps interferes with DNA extraction in other ways.

**Figure 6:**
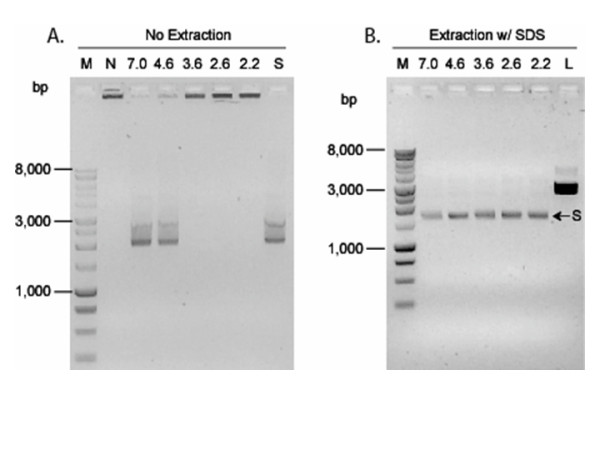
**Low pH restores the DNA binding and protection activities of heat-denatured Dps**. Heat-inactivated Dps (10 μg, 75°C, 15 min) was mixed with supercoiled pUC18 plasmid DNA (Dps:DNA (w/w) ratio of 33:1) and incubated for 1 h. The pH of the mixture was adjusted to the pH value indicated above each lane and incubated for 2 h at room temperature and analyzed by 1% agarose gel electrophoresis. Lane M, 1 kb DNA marker (Promega). Lane N, control experiment using native Dps protein, incubated at pH 7.0. Lane S, supercoiled pUC18 plasmid DNA. Lane L, *Hind*III linearized pUC18 plasmid. Agarose gels are visualized following staining with ethidium bromide. (A) No extraction step to purify DNA from protein. (B) DNA was extracted using chloroform:isoamyl alcohol (24:1) in the presence of 2% SDS.

Additional evidence that Dps-DNA complex formation may be affected by pH was gained from a negative control. When heat-denatured (75°C, 15 min) Dps was used as a control in experiments aimed at determining the role of Dps in plasmid DNA protection from acid damage, heat-denatured Dps did not bind to DNA at pH 7.0, as evidenced by the migration of pUC18 plasmid DNA in agarose gels (Fig. 6A). Similar results were observed with Dps-plasmid mixtures incubated at pH 4.6; however, the incubation of heat-denatured Dps at pH 3.6 or below restored Dps binding to DNA (Fig. 6A). This binding also protected DNA from acid damage (Fig. 6B). These results showed that in low-pH conditions, heat-denatured Dps regained its ability to bind and protect DNA from acid damage.

**Figure 7:**
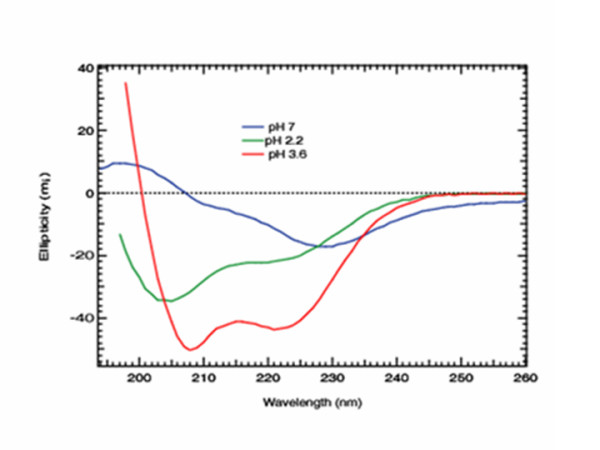
**CD spectra of Dps**. CD spectra of Dps revealed different secondary conformations at different pH. The spectrum for Dps at pH 3.6 is typical of secondary structures consisting primarily of α-helices.

The previous results suggested that Dps-DNA complex formation or stability was influenced by pH. Therefore, the secondary structure of Dps in low pH conditions was examined using circular dichroism spectroscopy (Fig. 7). The spectra showed that at pH 2.2 and 3.6, Dps secondary structure contained an alpha-helix conformation [29], approximately 54% at pH 3.6 and 23% at pH 2.2. An unrecognized spectrum was generated at pH 7.0 that was likely due to Dps aggregation [30]. Taken together, these three lines of evidence showed that pH influenced Dps-DNA interactions, at least between pH 3.6 and 2.2, and low pH appeared to enhance Dps association with DNA that contributed to its ability to physically protect and/or buffer DNA from acid damage.

## Discussion

Enteric bacteria encounter varied environments during host-to-host passage, including short-term exposure to the acidic conditions of the stomach during passage to their preferred niche in the colon. We have shown in a previous study that *dps *contributes to acid tolerance in the human pathogen *E. coli *O157:H7 by an unknown means [10]. The present study advances our understanding of the detrimental effects of acid stress by demonstrating that DNA damage occurred during exposure to acid stress and that the integrity of DNA was maintained through physical protection with Dps and by RecA-mediated repair.

Previous studies investigating the role of Dps in oxidative-stress protection found that the binding of Dps to DNA is not solely responsible for its ability to prevent damage. For instance, Dps-1 from *Deinococcus radiodurans *binds to DNA but does not provide protection from oxidative damage [31], while Dps from *Agrobacterium tumefaciens *does not bind to DNA but protects DNA from oxidative-stress damage [32]. This disparity in the mode of oxidative stress protection by Dps homologs is likely a consequence of the bi-modal protection of DNA described in *E. coli *where both the physical association and the ferroxidase center, which cages Fe^2+ ^ions and prevents formation of hydroxyl radicals by Fenton chemistry, protect DNA from oxidative damage [20]. To determine if iron influenced acid tolerance, parent and *dps *strains were pre-incubated for 30 min with 100 μM deferoxamine methanesulfonate (DFOM), an iron chelator, before being subjected to acid challenge. No statistically significant difference was observed in either the parent and *dps *strains incubated with or without DFOM (data no shown). These results suggested that iron and the ferroxidase center did not play a role in acid tolerance.

The analysis of chromosomal DNA extracted from *E. coli *O157:H7 exposed to pH 2.0 found signs of damage (formation of DNA fragments as evidenced by tailing) that increased with exposure time. Based upon these findings and as another approach to confirm the presence of DNA damage from acid stress, the role of RecA was investigated due to its role in recombinational repair of DNA. Chromosomal DNA from *recA *and *dps recA *mutants contained more strand breaks, as determined by results from *Bal*31 digestions, than either the *dps *mutant or the parent strains. Not surprisingly, this increase in acid-induced DNA damage in *recA *and *dps recA *mutants significantly reduced survival during acid stress. Notably, the acid tolerance of the *recA *mutant was significantly lower than that of the *dps *mutant and parent strain. These findings suggest that DNA repair might play a heretofore undervalued role in acid tolerance. Depurination and depyrimidination of DNA has been attributed to acidic pH. While both reactions occur at neutral pH in a temperature dependent manner, the rate constant of depurination/depyrimidination increases linearly with decreasing pH [4,33]. Therefore, acid stress will accelerate DNA damage due to the drop in internal pH regardless of the final cytoplasmic pH. Even when pH homeostasis systems are induced, like glutamate and arginine decarboxylases, the internal pH drops to 4.2–4.7 when acid challenged at ca. pH 2.5. In the absence of the respective amino acid substrates for these decarboxylases, internal pH drops further to around 3.6 [34]. In addition to accelerating the rate of depurination/depyrimidination of DNA, acid stress is likely to exert an additional effect on DNA damage by reducing the efficiency of RecA, since RecA activity has a pH optimum of 6.2 [35]. The lack of discernable differences in *Bal*31 digestion patterns of DNA between the *recA *and *dps recA *strains following acid challenge was reflected in the survival of the respective strains. Noticeably, the *recA *mutant exhibited lower survival than either *dps *or the parent strain, suggesting a critical role for DNA repair in response to acid stress. Other genes in the DNA-repair pathway, such as *recB *and *recD*, or in other DNA repair pathways, will be examined in future studies for their effects on acid tolerance. Also, it is probable that cross protection to acid occurs by exposure to other stresses that activate DNA repair pathways, such as brief exposure to UV irradiation, which will be examined to understand the integrated networks of stress tolerance in this pathogen.

Similar to the findings with chromosomal DNA from whole cells, *in vitro *studies demonstrated that plasmid DNA was protected from acid damage in the presence of Dps. To standardize *in vitro *assays, the Dps:DNA binding ratio (w/w) used was 33:1 (or ~1:1 mole/mole) and was set at this ratio based upon the hindrance of DNA migration into the agarose gel, although lower ratios provided some protection to the DNA from acid damage (data not shown). Prior reports estimate that in stationary phase, *E. coli *contains approximately 200,000 Dps molecules (19 kDa) per cell which equals approximately 3.8 × 10^6 ^kDa [14]. With an estimated genome size of 5.5 Mb for *E. coli *O157:H7 and 660 daltons per nucleotide pair, the genome is estimated at 3.6 × 10^6 ^kDa that results in a ratio of Dps:DNA *in vivo *of 1:1 (w/w), or 3:1 mole/mole. Several factors may contribute to the need for fewer Dps molecules to protect DNA in the *in vitro *condition than *in vivo*. First, not all the Dps molecules *in vivo *may be bound to DNA. Second, the presence of other DNA-binding proteins, like IHF, in stationary-phase cells that contribute to the nucleoid architecture and/or the protection of genomic DNA may affect how Dps interacts with the chromosome *in vivo *[15,36]. Finally, the *in vitro *data reported here may be a result of other factors specific to the experimental system, e.g. the use of Tris resuspension buffer (pH 6.3) containing EDTA, the plasmid DNA, or other unidentified conditions [21,27]. The optimal binding ratio of Dps:DNA reported here differs from a previous reported value of 8:1 (mole/mole) [30]. The discrepancy may be a result of differences in experimental set up, where Ceci *et al*. (2004) employed linear double-stranded DNA of 500 bp and we used super-coiled plasmid DNA 2,686 bp in size.

After incubation of the Dps-DNA mixture and subsequent exposure to acid, it was noted that the quantity of DNA recovered from acid exposed Dps-DNA complexes using non-denaturing extraction [phenol:chloroform (1:1)] decreased proportionately with a reduction in the pH. When denaturing conditions were used for extraction [chloroform:isoamyl alcohol (24:1) with 2% SDS], the quantity of DNA extracted did not change regardless of the challenge pH. These observations suggested that the association of Dps with DNA was influenced by pH. Additional support for the influence of low pH impacting Dps structure or interactions with DNA was obtained from control samples containing heat-denatured Dps. Heat denaturation (15 min, 75°C) of Dps nearly eliminated all binding of Dps to DNA at pH 4.6–7.0 (Fig. 6). However, incubation of heat-denatured Dps with DNA at pH 2.2–3.6 restored its ability to complex with DNA and to protect DNA from acid damage. These findings suggested that low pH facilitated changes in heat-denatured Dps that restored DNA- binding and protection activities.

Dps protein is believed to self-associate as dodecamers to establish a three-dimensional hexagonal structure in which the lysine-rich N-terminal regions of Dps subunits are involved in both DNA binding and Dps-Dps self aggregation [19,21,30]. Further, reports have shown that the Dps-DNA complex forms a coral reef structure [22], resulting in biocrystals in starved cells [19]. Since Dps has no DNA binding motifs and the surface of the Dps dodecamer is dominated by negative charges, Mg^2+ ^has been proposed to act as a bridge between Dps and negatively charged DNA [14,21,27]. Ceci et al. (2004) reported that both self-aggregation and DNA condensation required protonation of the N-terminus lysines (at least Lys-10), but the minimum pH tested in this study was 6.3. Thus, the lower pH values tested in our study provide additional data to support the idea that protonation of the N-terminus lysines and possibly other surface residues on the protein contribute to self-aggregation and/or DNA condensation. In DpsA and DpsB from *Lactococcus lactis*, the N-terminal regions are also known to form surface-exposed α-helices [37]. We demonstrated by circular dichroism (CD) spectroscopy that at low pH (3.6 and 2.2) Dps secondary structure contained an alpha-helix conformation. Collectively, results from this study demonstrated that pH influences Dps-DNA complex formation and possibly structure, which is important to the protection of cellular DNA from the deleterious effects of acid damage.

Dps is known to participate in acid stress protection in *E. coli*, but it was unknown whether this occurs through a direct or an indirect mechanism. Results from this study showed that Dps protected DNA by direct interaction. Studies of chromosomal DNA from acid-stressed cells revealed more extensive DNA damage in *dps *and *recA *mutants. Results also suggested that the decrease in cytoplasmic pH could also influence the formation and/or stability of Dps-DNA complexes. Since Dps is abundant in stationary phase cells, and since the binding of Dps to DNA under acidic conditions is rapid and energy-independent, Dps is well placed to combat acid stress. Both Dps and the *recA*-mediated DNA repair pathway contribute to the maintenance of DNA integrity under acid stress conditions. Investigation into the precise interplay between DNA protection and damage repair pathways during acid stress are underway to gain additional insight on acid tolerance in human intestinal pathogens, like *E. coli *O157:H7.

## Authors' contributions

KCJ and KFH constructed and confirmed mutants, collected and analyzed data, and contributed to the drafting of this manuscript. DJB and JJB participated in the collection of data on acid tolerance, data interpretation, and the drafting of the manuscript. CWK developed the project concept, analyzed data, coordinated project activities, and drafted the manuscript.
